# Multi-Layer Picture of Neurodegenerative Diseases: Lessons from the Use of Big Data through Artificial Intelligence

**DOI:** 10.3390/jpm11040280

**Published:** 2021-04-07

**Authors:** Andrea Termine, Carlo Fabrizio, Claudia Strafella, Valerio Caputo, Laura Petrosini, Carlo Caltagirone, Emiliano Giardina, Raffaella Cascella

**Affiliations:** 1 IRCCS Santa Lucia Foundation, Genomic Medicine Laboratory UILDM, 00179 Rome, Italy; andreatermine544@gmail.com (A.T.); claudia.strafella@gmail.com (C.S.); v.caputo91@gmail.com (V.C.); raffaella.cascella@gmail.com (R.C.); 2 IRCCS Santa Lucia Foundation, Laboratory of Experimental and Behavioral Neurophysiology, 00143 Rome, Italy; carlo.fabrizio217@gmail.com (C.F.); laura.petrosini@uniroma1.it (L.P.); 3 Department of Biomedicine and Prevention, Tor Vergata University of Rome, 00133 Rome, Italy; 4 IRCCS Santa Lucia Foundation, Department of Clinical and Behavioral Neurology, 00179 Rome, Italy; c.caltagirone@hsantalucia.it; 5 UILDM Lazio ONLUS Foundation, Department of Biomedicine and Prevention, Tor Vergata University, 00133 Rome, Italy; 6 Department of Biomedical Sciences, Catholic University Our Lady of Good Counsel, 1000 Tirana, Albania

**Keywords:** artificial intelligence, big data, deep learning, neurodegenerative diseases, precision medicine

## Abstract

In the big data era, artificial intelligence techniques have been applied to tackle traditional issues in the study of neurodegenerative diseases. Despite the progress made in understanding the complex (epi)genetics signatures underlying neurodegenerative disorders, performing early diagnosis and developing drug repurposing strategies remain serious challenges for such conditions. In this context, the integration of multi-omics, neuroimaging, and electronic health records data can be exploited using deep learning methods to provide the most accurate representation of patients possible. Deep learning allows researchers to find multi-modal biomarkers to develop more effective and personalized treatments, early diagnosis tools, as well as useful information for drug discovering and repurposing in neurodegenerative pathologies. In this review, we will describe how relevant studies have been able to demonstrate the potential of deep learning to enhance the knowledge of neurodegenerative disorders such as Alzheimer’s and Parkinson’s diseases through the integration of all sources of biomedical data.

## 1. Introduction

Neuronal degeneration is a common cause of morbidity and cognitive impairment in the elderly [1]. Neurodegenerative Diseases (ND) are a large group of neurological disorders with heterogeneous clinical and pathological expressions, affecting specific subsets of neurons in specific functional anatomic systems, placing a considerable burden on an increasingly aging society [2]. ND are broadly identified as proteinopathies due to conformational changes affecting protein functionality, thereby causing toxicity or losing their physiological function: misfolded proteins start to aggregate resulting in neurotoxicity [1,3]. ND are characterized by a high level of heterogeneity and complexity in terms of clinical presentation and etiology because of the interaction of genetic, lifestyle, and environmental factors [3,4,5,6]. Notably, the heterogeneity of ND is a key confounding factor that complicates the understanding of disease mechanisms and the identification of treatments. Case-control cohorts often include multiple phenotypes on distinct disease trajectories or rely on models that only account for a few features of the central nervous system at a time, which has been reductive for complex diseases [7,8,9]. Alzheimer’s (AD) and Parkinson’s (PD) diseases are two of the most frequent and heterogeneous pathologies among all the complex neurodegenerative proteinopathies, affecting 24 and 6.1 million people worldwide, respectively [3,7,10]. Both disorders include hereditary Mendelian forms, caused by mutations in single genes and complex sporadic forms characterized by polymorphisms in multiple genes that interact with environmental, epigenetic, and transcriptomic signatures in determining the heterogeneity and the differential susceptibility to disease [4,11]. To date, the identification of AD and PD therapeutic targets and in vivo biomarkers for early diagnosis is still challenging, because of the existence of different disease subtypes (phenotypic heterogeneity) and stages of disease (temporal heterogeneity) [8]. Driven first by genomic studies and more recently by transcriptomic and epigenomic studies, a large volume of data has been rapidly produced to tackle this heterogeneity. In the perspective of ND as a big data issue, such diverse observations could be pulled together to provide a personalized, multi-layer representation of patients, which considers the complex heterogeneity of the disease and the availability of effective diagnostic criteria and drug development deliverables. In this context, computational modeling and simulation represented key components of the scientific method in which both reductionist and holistic approaches are not treated as separate fields but as convergent and cross-supportive paths [7,8,9,12]. Therefore, this review aims to analyze the rapidly evolving techniques for data integration of multi-omics, clinical, and neuroimaging data discussing their role in a precision medicine framework [4,13,14]. Deep Learning (DL) techniques will be discussed with relevant examples concerning the identification of biomarkers for prognosis, early diagnosis, and assessment of symptoms, considering observations on handwritings, speeches, and movement dynamics. A specific focus will be given to articles building and analyzing a multi-layer representation of subjects, showing off the advantages offered by big data integration. Finally, publicly available databases collecting multiple sources of biomedical information for ND will be reviewed.

### Literature Research

Relevant applications of Artificial Intelligence (AI) techniques to ND have been selected from specific research queries on bibliographic search engines such as PubMed, Google Scholar, and Dimensions.ai. “Artificial Intelligence”, “Deep Learning”, “Machine Learning” were used as keywords to identify AI-related articles, in combination with “neurodegenerative”, “Alzheimer” or “Parkinson” to address the pathology. Ultimately, these were combined with “speech”, “segmentation”, “handwriting”, “voice”, “movement”, “multi-omics”, “EHR” or “data integration” to retrieve literature publications exploiting the related data types. Titles and abstracts were checked to identify relevant articles that were finally included in this review. Notably, we decided to include experiments with reported accuracy below the 95% threshold, which is the cut-off meet minimum Medical Diagnosis Treatment (MDT) standards and pass a ‘medical Turing Test’ [15], because we wanted to represent the state of the art of DL and ML applications in the field of neurodegenerative diseases data integration.

## 2. Basics of Machine Learning and Deep Learning

Machine Learning (ML) encompasses a collection of data analysis techniques aiming to generate predictive models from multi-dimensional datasets [16,17]. The advantages of ML come from its ability to learn from previous data to make accurate predictions on new data in both supervised and unsupervised contexts, with reduced or absent assumptions [17]. The focus of unsupervised methods is to learn patterns in the features of unlabeled data, while supervised methods aim to discover the relationship between input features and a target attribute, e.g., an MRI brain scan from a patient labeled as Alzheimer’s [16].

**Figure 1:**
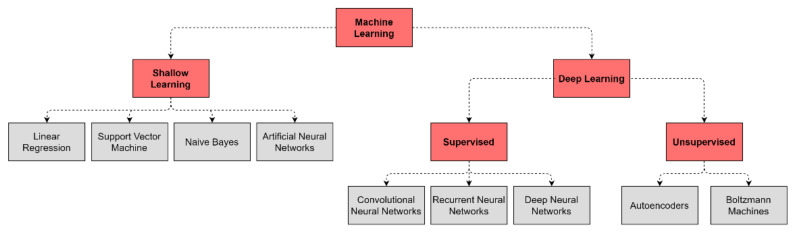
Common algorithm types for ML and DL employed in ND biomedical research.

DL differs from the traditional ML algorithms applied in biomedical classification tasks, such as linear or logistic regression, Support Vector Machine (SVM), and naive Bayes classifier due to its ability to cope with the complexity and volume of multi-layer data (Figure 1) [16,18]. DL models are based on Artificial Neural Networks (ANN) that are loosely inspired by human brain networks and a typical DL architecture is organized in layers of computational units known as “neurons” [16]. Traditional ML algorithms and basic ANN are considered shallow learners, learning from data described by pre-defined features or by expert-based descriptors. These shallow learners produced significant progress both in medicine and multi-omics fields and led to the identification of multigene signatures potentially involved in disease onset and progression in ND [18]. However, the advent of Deep Neural Networks (DNNs) outperformed shallow learners, as DNNs can combine multiple hidden layers to provide a deeper and more comprehensive representation of data and allow the exploration of complex interrelationships between genetics, biochemistry, histology, and disease status. Notably, these DL methods can extract features automatically from raw data with little or no preprocessing, overcoming manual features engineering (Table 1) [16,18].

## 3. Artificial Intelligence in Neurology

**Figure 2:**
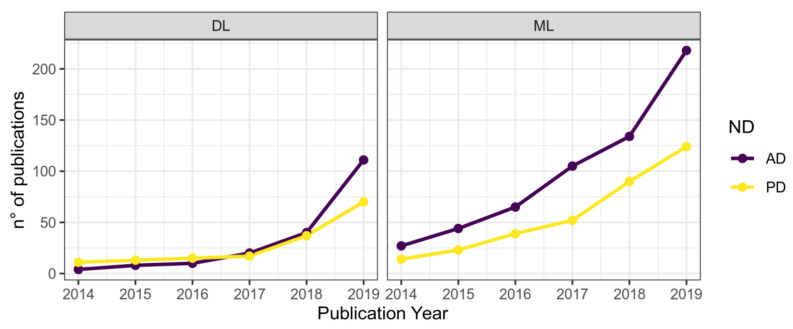
Number of publications in ML or DL fields by year and ND. Data were retrieved on dimensions.ai using Alzheimer’s or Parkinson’s diseases and deep learning or machine learning as keywords to search in title and abstract. Results were limited to “article” as Publication Type.

AI allows for automated data interpretation and decision-making. The peculiarity of AI is to be able to learn from data to acquire knowledge, represent and process information related to the task it has to perform, thereby overcoming the difficulty to assimilate and extract valuable information from large datasets. Thus, AI can be used as a powerful tool in the elaboration of biomedical data for the development of predictive models. One of the most relevant data sources for AI comes from the biomedical field, and the ability of DL—one of AI’s most important branches, alongside ML—to automatically learn complex representations from data is showing to be particularly promising to help ND research and clinical management [18,23]. Nowadays, the number of publications in the ND research area employing DL techniques (Table 1) and other ML algorithms is constantly increasing (Figure 2). Classification and segmentation of neuroimaging data is a traditional subdomain of DL methods application, stating the high-dimensional nature of neuroimaging data that is highly suitable for AI intervention, and relevant application examples are presented below. Afterward, it will be shown how observations on handwritings, speeches, and movement dynamics can be used to support symptoms and diagnostic assessment. In the subsequent section, we discuss the usefulness of merging multiple data types, including multi-omics, clinical, and neuroimaging data to obtain a holistic representation of subjects.

### 3.1. Neuroimaging Classification and Segmentation

Biomedical imaging is a traditional field of application for DL architectures. To date, classification and segmentation tasks on neuroimaging data have been greatly improved by employing AI techniques [18,23]. DL models can be applied to classify ND stages or sub phenotypes. As a representative application in AD, a CNN-based approach has been implemented by Ramzan and colleagues on resting-state fMRI of 138 AD subjects from the Alzheimer’s Disease Neuroimaging Initiative (ADNI) database. The final model achieved an average accuracy of 97.92% on the test set, classifying subjects among six different stages of AD including Cognitively Normal (CN), Significant Memory Concern (SMC), Early Mild Cognitive Impairment (EMCI), Mild Cognitive Impairment (MCI), Late Mild Cognitive Impairment (LMCI), and AD [24]. A noteworthy study focused on the detection of PD from volumetric T1-weighted MRI scans used a 3D CNN to classify patients over control subjects (CS). They used data from the PPMI database [25] (described in Section 5.) and obtained an average recall, precision, and F1-score of 0.94, 0.93, and 0.94, respectively. Their model demonstrated to be good enough to not misclassify any PD subject [26]. CNNs can also be applied in the segmentation task to quantify structural changes in brain shape, volume, and thickness that may be related to neurodegeneration [18,27]. As the assessments of the brainstem and hippocampal volumes are known to be crucial tasks in the study of ND, a 2D CNN was recently used to predict the number of voxels attributed to the hippocampus [28]. Meanwhile, an automated sub-cortical brain structure segmentation approach based on a CNN architecture outperformed state-of-the-art techniques such as Free Surfer on the Internet Brain Segmentation Repository (IBSR 18) dataset [29]. A DL-based hippocampus segmentation framework embedding statistical shape of the hippocampus as “context information” into DNN was proposed and tested on image data of AD, MCI, and CN subjects from two cohorts from ADNI and AddNeuroMed, leading to improved segmentation accuracy in cross-cohort validation [30]. Notably, DL can be used as a feature extractor before classification tasks reducing the need for rigid segmentation in preprocessing: a multiple dense CNN was used on an ADNI dataset, including 199 AD patients, 403 MCI, and 229 CN. Experimental results showed that the proposed method achieves an accuracy of 89.5% for AD vs. CN classification, and an accuracy of 73.8% for MCI vs. CN classification [31]. Moreover, another CNN model based on transfer learning was used as a feature extractor in a multi-class discrimination task on the ADNI database, achieving an overall accuracy of 95.73% on the validation set [32]. Transfer learning is defined as the ability of a system to recognize and employ the knowledge learned in a previous source domain to a novel task and it can be implemented in segmentation to reduce the need for many annotated samples to perform the training task [27]. Transfer learning is characterized by some limitations because objects in biomedical images may have very different appearances and sizes so transfer learning from the models with huge variations in organ appearance may not reduce the segmentation result [27]. Overall, AI flexibility in learning complex and abstract representations of neuroanatomical data through nonlinear transformations is particularly promising since it can greatly improve the knowledge of the aging brain and its response to several concurrent pathological processes.

### 3.2. Clinical Records Investigation

**Figure 3:**
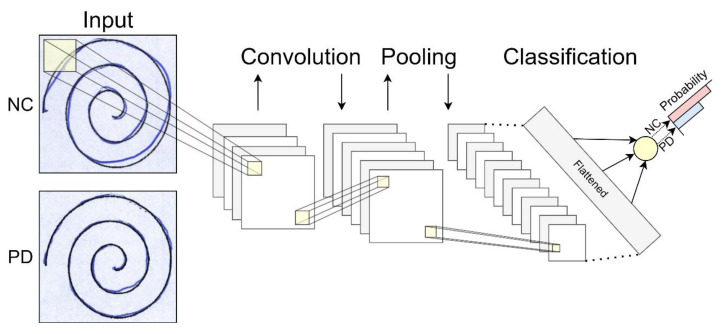
Deep Learning handwritings classification. CN and PD handwritings are hard to distinguish if not trained to. A CNN can be made capable of classifying patients and controls upon almost imperceptible changes in subjects’ drawings. Convolution and pooling operations process input data to extract relevant features from the images, allowing detection of group differences. Spirals images were taken from the NewHandPD dataset [35], available at http://wwwp.fc.unesp.br/~papa/pub/datasets/Handpd/, accessed on 5 January 2021.

In addition to widespread research on DL applications for image classification and segmentation, researchers have applied AI to several neurological and general medical data. ML and DL techniques have been exploited to support clinical expertise analyzing handwritings, voice recordings, and movement registrations. Handwriting deterioration is one of the most typical clinical hallmarks of PD and the identification of distinctive handwriting features can help to build a predictive model for PD classification [33]. Drotár and colleagues [34] collected handwriting samples from a sample of 37 PD Czech patients on medication and 38 matched controls. They extracted relevant features from data using statistical methods and fed them to an SVM with a Radial Basis Function kernel, achieving 88.1% as the highest accuracy in classifying PD patients [34]. Another interesting usage of patients’ handwriting is shown in a recent study by Pereira and colleagues [33]. Using an electronic pen to map handwriting dynamics by PD patients into computer images, researchers collected data to be analyzed by a CNN. The authors obtained a final accuracy of about 95% in classifying PD patients and healthy controls, supporting the employment of a DL-based approach to aid PD diagnosis. Interestingly, they showed the goodness of the model in distinguishing healthy controls from patients with early-stage PD. Their CNN has been challenged in classifying data from eight manually-selected patients with very similar traces to healthy individuals. The accuracy rate above 94% proved it to be robust enough to detect almost imperceptible changes between the two groups’ handwritings (Figure 3) [33].

These approaches can be considered as alternative or complementary to others, such as speech or movement-based discriminant analyses. Various methods have been presented for analyzing patients’ speech and movement recordings. As an example, Berus and colleagues exploited speech recordings data from 20 PD and 20 CS [36]. Recordings were taken during a medical examination while subjects were reading or saying certain numbers or words, for a total of 26 recordings per subject. A fine-tuned ANNs ensemble algorithm was trained to classify each voice sample for each subject. A class was finally attributed by the majority voting of each ANN constituting the ensemble. Their algorithm achieved a test accuracy, sensitivity, and specificity of 86.47%, 88.91%, and 84.02%, respectively [36]. Another possible use of voice recordings is presented in a very recent paper by Al-Hameed and colleagues [37], where the authors showed how it is possible to discriminate between patients reporting cognitive concerns attributable to ND or Functional Memory Disorder (FMD, i.e., subjective memory concerns unassociated with objective cognitive deficits or risk of progression) by analyzing acoustic features extracted from speech recordings. Recordings data from subjects’ clinical conversations with the neurologist during the diagnosis assessment were processed for feature extraction and selection and then used to train five different ML classifiers to differentiate between the two classes. This method achieved an average accuracy of 96.2%, proving that the discriminative power of purely acoustic approaches could be integrated into diagnostic workflows for patients with memory concerns. Interestingly, this method does not require automatic speech recognition and understanding because it relies only on acoustic features obtainable from recordings processing [37].

PD patients manifest motor symptoms such as bradykinesia, tremor, and posture alteration, and clinical observations can be taken from their characteristic gait. Gait disorders in PD are characterized by spatial and temporal dysfunctions and Freezing Of Gait (FOG) is one of the most debilitating motor symptoms in PD. DL algorithms can be implemented in automatic systems of FOG detection, as recently demonstrated [38]. In this paper, the researchers analyzed wearable sensor data with a CNN to automatically detect when a FOG episode would occur, achieving 89% accuracy. This study presents the first method of FOG detection on home environments based on DL techniques, showing outperforming results over other previous automatic methods and possibly improving the medical monitoring of FOG’s evolution in PD patients. Finally, this tool can also be beneficial to evaluate the effects of drugs during clinical trials [38].

## 4. Big Data Integration

As 21st-century biomedicine goes through the big data era, the production of a wide variety of biomedical data gets simpler and faster [7,23]. To face the data volume and heterogeneity increase, data sharing initiatives were encouraged by funding agencies and scientific journals, and publicly available repositories and databases were established [9,39]. However, standardized protocols for cross-platform interoperability, data management strategies, and common workflows for data sharing and analysis lagged an increasingly faster data production, hurting model deployment and insights generation [7]. Multi-omics and EHRs data isolation still pose considerable challenges for researchers’ abilities to access, integrate, and model often noisy, complex, and high-dimensional data [7,8,17,23,39]. In the next section, data accession and integration strategies both for data management and analytics will be discussed, introducing multi-omics and EHRs data. Finally, a list of curated databases for ND will be presented and local or international consortia initiatives aiming to maximize both sample collection and data generation will be reviewed.

### 4.1. Multi-Omics

**Figure 4:**
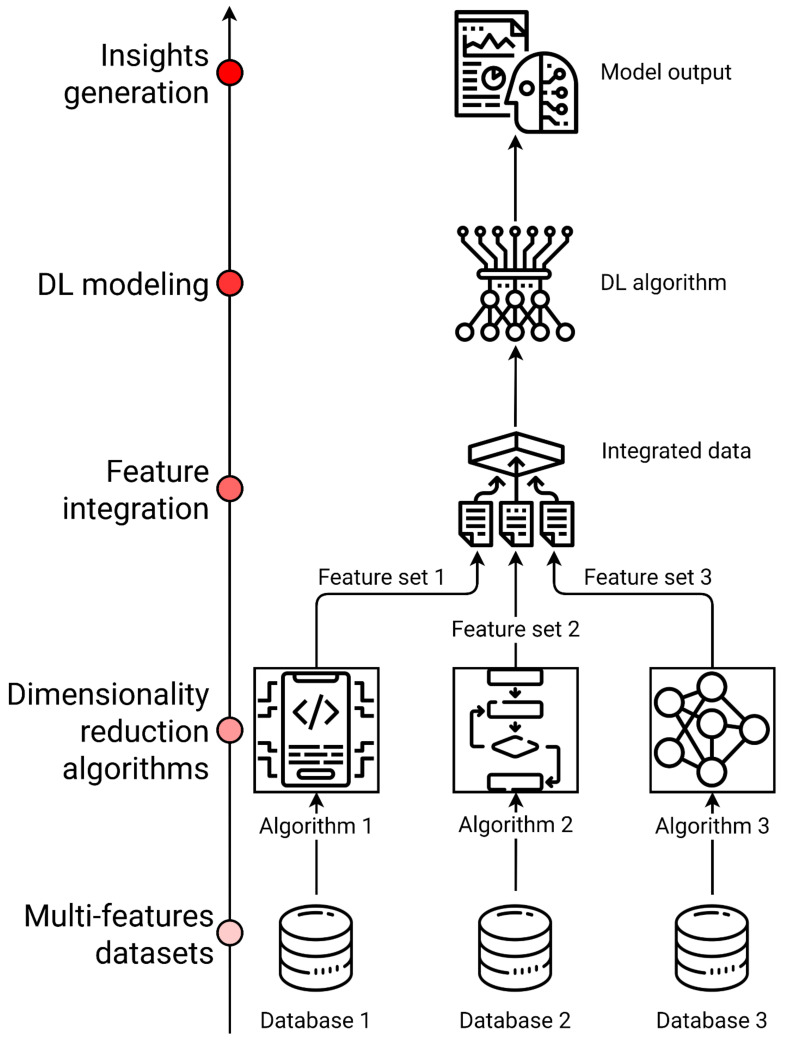
A DL workflow implementing dimensionality reduction strategies to integrate large and heterogeneous datasets. Dimensionality reduction algorithms can be applied to standard multi-omics data, integrating different features from the same set of observations or obtaining one outcome variable from different layers of biological systems.

Biological systems consist of several molecular features such as genes, proteins, as well as interactions between those components. Omics refers to the comprehensive characterization and quantification of these molecules, grouped according to their structural or functional similarities [17,40]. Multi-omics data integration combines information from different layers of omics data to understand how different biological systems interact at a molecular level [17,23]. This is relevant in ND such as AD and PD, where a multifactorial etiology is usually combined with heterogeneous clinical pictures and mixed pathologies [12]. Multi-omics data can be classified as (1) multi-feature data when the same set of samples presents several distinct feature sets, or (2) multi-relational data with different features and different sample sets in the same phenomenon or system. However, some variation in data architecture is possible, such as (3) multi-class data with different groups of samples measured by the same feature set and (4) tensor data measuring the same set of objects by the same set of features in different conditions [41]. Data-driven analysis of multi-omics data in ND can be performed to screen for potential biomarkers and druggable targets or to identify sub phenotypes through clusterization methods. Furthermore, the interactions among different sets of features could be crucial to understand the pathogenic pathways underlying different disease phenotypes, each one defined by its biomarkers as a phenotypic subtype with its own suitable personalized treatment [42]. Nevertheless, data integration of multi-omics data is still a major challenge in precision medicine, since omics analyses are impeded by high analytical variance and limitations in experimental design, resulting in a low signal-to-noise ratio [23]. Moreover, ND complex presentation is also subjected to temporal heterogeneity and individual variance in terms of biological measures and technical error [7,8,12,23]. To this purpose, different strategies have been proposed to produce trustworthy results and insights and to manage single and multi-omics experimental design and analysis issues. Integration algorithms can be organized in workflows both for integrated or orthogonal omics datasets [7]. Dimensionality reduction methods are a set of ML multivariate techniques for feature extraction based on matrix factorization and while it is often challenging to combine features of multiple omics data, new features generated by these methods can easily be combined for every class of multi-omics data (Figure 4) [23,41].

### 4.2. Electronic Health Records (EHRs)

Data isolation represents one of the major issues in big data analytics and for healthcare entities trying to construct EHRs protocols and databases. Healthcare data are typically dispersed across various medical systems located at multiple sites and many of these systems are not interconnected, constraining the data into isolated silos and contributing to the increase in the expenses of institutions [43]. EHRs contain patients’ demographics along with clinical measurements, interventions, clinical laboratory tests, and medical data, thereby constituting one of the pillars of big data in the biomedical field [44]. EHRs data are both structured and unstructured, the former being represented by diagnostic codes and laboratory test outputs, the latter being represented by physician annotations about patients’ status. Analysis of this kind of data is not feasible using classical statistical methods and more sophisticated techniques (such as DL) are required. To fully exploit the big data potential, all data sources must be considered to avoid discarding data due to their being unstructured. Free-text clinical notes in the EHRs, which can only be analyzed with a DL approach, can give useful information about the patients and can improve the accuracy of analytical results [23,45]. Data isolation prevents healthcare organizations from leveraging the latest Information Technologies (IT) innovations (such as data processing and cloud computing), which can help to improve care and significantly reduce costs [43]. Similar to what happened in multi-omics data management, data standards have been developed to overcome healthcare information sharing and interoperability issues across different healthcare systems [39,43]. Fast Health Interoperability Resources (FHIR) is a modern healthcare data format and exchange standards widely used to encode EHRs data [46]. FHIR implements an application programming interface with HTTP-based RESTful protocols and enables interoperable communication and information sharing between various healthcare systems, enabling their integration with mobile devices and cloud platforms. FHIR data have a well-defined structure, covering a variety of healthcare aspects including clinical, administration, financial, reporting studies. These data are called “resources” and they are easily extensible to cover non-standard use-cases. FHIR features and flexibility is ideal to effectively generate EHR datasets to be integrated with other omics data [23,43]. FHIR coded data, images, and other features processed with different standards can be integrated with cloud platforms, such as Google Health API or Amazon Comprehend Medical. Successful and standardized integration of big data in the healthcare system can be applied to real-time healthcare analytics to improve care service quality and costs [47,48]. Such approaches of continuously using newly generated data in ML applications would be interesting even in other contexts, such as in pandemic situations.

### 4.3. Artificial Intelligence Applications on ND Multi-Omics and Clinical Data Integration

Researchers exploiting biomedical big data for ND aim to empower clinical efficiency by combining various sources of information such as multi-omics, EHRs, and medical imaging (e.g., MRI) data, building a holistic representation of patients. DL models can be used as a cutting-edge data analysis technique to find patterns in a patient’s broad-scope view. This kind of approach can be hypothesis-free, exploring data in search of explanations for differences between groups instead of being hypothesis-driven as classical experiments [49,50]. By building the most accurate representation of patients possible through the integration of all sources of biomedical data, DL allows researchers to find multi-modal biomarkers to develop more effective and personalized treatments, early diagnosis tools, as well as useful information for drug discovering and repurposing [51]. Along with neuroimaging data, EHRs can provide useful information when AI takes the field. De-identified data from the PPMI database was used for the identification of PD subtypes [52]. The authors used a Long-Short Term Memory (LSTM) network to analyze patient data referred to six years of measurements on potential PD progression markers, including clinical features, imaging, bio-specimen measures, and demographics. LSTM can analyze time series data, allowing the authors to represent patients by considering value progression for the available features. The analysis brought to identify three PD subtypes with distinct patterns of progression, demonstrating heterogeneous characteristics within PD patients’ features. The integration of biomarkers and clinical data for DL application showed that the disease progression rates, and the baseline severities are not necessarily associated and that motor and non-motor symptoms are not necessarily correlated [52]. This experiment is a good example of how DL techniques enable the management of integrated multi-domain data. Another application of a multi-modal DL approach was used to predict MCI to AD progression [53]. ADNI longitudinal data from cerebrospinal fluid biomarkers, neuroimaging, cognitive performance, and demographics were integrated and analyzed through a multimodal Recurrent Neural Network (RNN). This method allows integrating multiple domain data for multiple time points. Their results show that DL models perform better on integrated data than on separated single modality data, achieving a higher prediction accuracy. This approach could potentially identify people who might benefit the most from a clinical trial and assess risk stratification within clinical trials [53]. Integration of multi-omics heterogeneous data was used to predict AD diagnosis [54]. The authors implemented a DNN to predict AD using large-scale gene expression and DNA methylation data from prefrontal region tissue of different individuals diagnosed with late-onset AD. Results showed higher accuracy in predicting AD with multi-omics integrated data rather than with single-omics data. The authors also compare accuracy results from conventional ML methods with their proposed DL method, observing an improved predictive performance [54]. Currently, the use of DL methods on multi-omics integrated data is far more common in cancer research than in ND research, as fewer studies report the use of these methods in this area [55]. Overall, data integration yields better classification and prediction results in almost every field where it is applied and is standing as the next level in biomedical research [23,41,56].

## 5. Databases

The adoption of academic and industry-wide data standards is a key element to enable large-scale experimental data integration opportunities [23]. Public availability of datasets is growing in all disciplines and the Findable, Accessible, Interoperable, Reusable (FAIR) principles have been proposed to promote good scientific practices for data sharing initiatives, while databases aggregators such as OmicsDI started to monitor repositories to facilitate discovering and linking of public omics datasets [39,57]. To have a comprehensive overview of complex ND and trace their underlying pathogenesis mechanisms and progression, different biomedical data needs to be integrated for modeling and pattern recognition. A list of major available databases where researchers can retrieve data to test their hypotheses and generate novel insights is reported in Table 2. The Parkinson Progression Marker Initiative (PPMI) is an international and multi-center study that collects data from PD patients for future biomarker discovery and personalized PD therapy. Interested researchers can download de-identified clinical, biomarker, and imaging data, including raw and processed MRI and SPECT images [25]. AD and related pathologies data can be found in the NIA Genetics of Alzheimer’s Disease Data Storage Site (NIAGADS). It is funded by the National Institute on Aging and provides access to multi-omics data from AD genetics projects [58]. One of the most interesting initiatives for ND data sharing is the Global Alzheimer’s Association Interactive Network (GAAIN), which federates more than 50 data partners and gathers data from more than 450,000 subjects, to improve the understanding, treatment, and preventative measures for AD [59]. Other databases such as the Alzheimer’s Disease Neuroimaging Initiative (ADNI) have made AD data publicly available upon standardization of data acquisition protocols for researchers to retrieve clinical, imaging, and omics data [60]. This initiative was putting aside the need for years-long data collection, facilitating and speeding up hypotheses testing. Nevertheless, data access is restricted by data use agreements requiring ADNI to be cited in manuscripts and prohibiting data redistribution [61]. GAAIN is instead a virtual community for sharing AD data, which is stored in independently operated repositories around the world, aiming to offer a data homogenization service to the scientific community [59]. GAAIN offers the possibility to download data mapped to its data-sharing schema, allowing time-saving in interpreting different terminologies and nomenclatures used by each data repository [61]. Another interesting data source is the Swedish study Bio FINDER, which aims to discover the key pathological mechanisms in ND by analyzing various sources of data such as neuroimaging, biospecimen, and clinical examinations. Data is not publicly available but can be requested for download. Moreover, as non-specific databases, including ND data, there are Gene Expression Omnibus (GEO) and UK Biobank, containing clinical and omics data for a wide range of health-related outcomes [62,63]. Another novel initiative with the main goal of providing a multi-layer picture of ND patients is the Italian IRCCS Network of Neuroscience and Neurorehabilitation, which encourages scientific research and translational technologies for improving diagnosis, treatment, rehabilitation, and prevention of neurodegenerative disorders [4,64]. In addition, the network is also working on providing remote motor and cognitive neuro-telerehabilitation treatments finalized to facilitate the access of patients to personalized healthcare approaches, provide a continuity of care, and adequate monitoring strategies [64]. Interested researchers can query the websites to find datasets fulfilling their needs. With many available databases providing digital data from ND patients, it is possible to collect big biomedical datasets. Studies integrating data from various sources aim to obtain a holistic description of ND patients’ characteristics and analyzing it using the best-suited techniques may lead to novel patterns identification in disease mechanisms.

## 6. Challenges and Limitations for AI Techniques in ND Research

**Figure 5:**
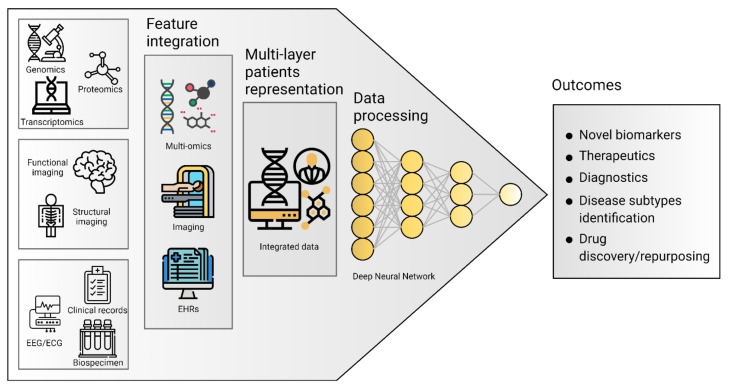
Multi-layer picture of neurodegenerative diseases. Separated data can be integrated to obtain a holistic representation of patients. Artificial intelligence techniques application for data processing leads to useful findings in ND research, clinical management, and personalized treatment development.

In the era of big data, the availability of biomedical information has exponentially increased, leading to technical and theoretical advances in data management, standardization, and analysis [66,67,68]. High-throughput technologies for genomic, transcriptomic, proteomic, and metabolomic analyses were accommodated in a network medicine framework focused on molecular and genetic interactions, biomarkers of disease, and therapeutic target discovery [40,69]. However, developing a comprehensive, holistic representation of patients with ND may require omics data to be merged with many other sources of information, such as EHRs, medical imaging, and wearable sensors data [23,50]. Therefore, multi-layer data integration is necessary to achieve a precision medicine approach, which is a unique opportunity to greatly improve healthcare quality and research outcomes in neurodegenerative pathologies for the identification of personalized treatments (Figure 5) [41,56,70]. As previously discussed in this review (Section 4 and Section 4.2), updated health informatics and data science workflows with a renewed data management policy are required to condense biomedical data vectors into an easily interpretable and translationally relevant form [7]. Data isolation in silos of non-communicating medical systems was discussed for EHRs, as it represents one of the major issues of the big data era, also affecting ND research. Only a few consortia initiatives have the resources to start collecting data with a multi-omics or a personalized medicine approach in their mind, leading to a multitude of isolated, low inter-operative datasets [7,9]. The adoption of FAIR principles and other standardization and monitoring processes such as OmicsDI will help to develop common ontologies and uniform data labels [39,57], while novel data-sharing initiatives with a defined big data architecture in mind, such as the National Virtual Institute for the investigation of Parkinson Disease in the Italian IRCCS Network of Neuroscience and Neurorehabilitation are starting to collect data in ND [4,64]. These new data sharing and encoding protocols are starting to shape a new direction in the biomedical field, and many authors suggest that these initiatives will become increasingly used as data volume and variety rapidly increases [7]. The implementation of a precision medicine approach in ND requires complementing classical case-control studies on less frequent diseases with community-based studies that are ideal for common neuropathologies [12]. Community design studies produce data that can be repurposed in multiple ways to look at specific outcomes, to derive new outcome measures, or to assess the interaction between many biological systems. As we progressively approach a holistic representation of the patients through an increasing volume, velocity, and variety of data generation, DL methods are being used to integrate and model those high-dimensional datasets [23,41,50]. Neural network architectures are flexible instruments uniquely allowing for labeled and unlabeled data processing and analysis. They can be used in the data integration phase as dimensionality reduction/feature extractor tools, and they are especially suited to leveraging large amounts of data from high-throughput omics studies or medical imaging. Notably, only DL has the potential to integrate the entire medical record, including physicians’ free-text notes [23]. Several limitations to DL implementation in personalized medicine research are being addressed, such as reduced sample size and reproducibility issues [50]. As an example, Semi-Supervised Learning (SSL) algorithms work both with mixed labeled and unlabeled data points, sometimes achieving a better performance than a fully supervised approach because the model can learn from a much larger set [17]. Another relevant issue in this field is the reproducibility of other studies and the implementation of other’s AI models. This is due to the lack of open-source implementations provided by authors and the difficulty of re-implementing a network in a different library. Automated code extraction from published papers is a scraping method enabled by DLPaper2Code to address reproducibility issues for DL architectures and it can be integrated into well-known DL frameworks [71]. Traditional DL issues, such as overfitting and interpretability represent common challenges for the development of reliable models. A model overfits the training data when it describes features that arise from noise or variance in the data, rather than the underlying distribution from which the data were drawn. Overfitting usually leads to loss of accuracy on out-of-sample data [72]. Overfitting is usually addressed using regularization methods or implicit/explicit feature selection techniques [73,74]. Cross-validation (CV) is a process for creating a distribution of pairs of training and test sets out of a single dataset. CV techniques such as hold-out and k-fold cross-validations have become industry standards, preventing the risk of overtraining. In k-fold CV, the data are partitioned into k subsets, each called a fold. The learning algorithm is then applied k times, each time using the union of all subsets other than the one left out, which will be used as a test set [72]. Moreover, DL models are commonly characterized by interpretability issues, reducing their potential as insights generators for clinicians and researchers [75]. To address this issue, several methods have been developed to understand how a DL architecture solves a regression or a classification problem [76,77,78]. Finally, data sparseness in computer-aided medical diagnosis and treatment still represents an unresolved challenge for machine diagnosticians, undermining AI diagnostic efficiency [15]. Calculations showed that the sparseness of actual symptom-treatments sets based on ICD-10 in the space of all possible sets is astronomical, thereby requiring to provide AI with more “functional” information, such as domain-specific medical reasoning processes and policies based on heuristic-driven search methods derived from human diagnostician methods [15].

## 7. Conclusions and Future Directions

In this work, we reviewed how AI can be applied to biomedical big data for ND research. After a brief introduction to ML and DL basics, we went through some notable AI applications on the most important biomedical data kinds. We have seen how neuroimaging, EHRs, and multi-omics data permit us to obtain better classification results when integrated together in constituting a unified representation for patients. Databases offering large-scale experimental data integration opportunities have been reviewed. Ultimately, big data integration is showing to be the next level in biomedical research, offering many advantages despite the limitations of such an approach, discussed in Section 6. Creating straightforward and interpretable DL models is a challenge for AI research in the healthcare field and several authors have attempted to address it [50]. A very interesting model for AD big data analytics is BHARAT, an application for integrated data manipulation, storage, and processing. BHARAT integrates brain structural, neurochemical, and behavioral data from magnetic resonance imaging, magnetic resonance spectroscopy, and neuropsychological testing, providing feature selection and ensemble-based classification. This framework’s focus is not only on AD classification through DL methods, but also on determining relevant information originating from the analysis of multi-modal integrated data, such as early diagnostic biomarkers for AD pathogenesis [79]. Most of the biomedical research fields will benefit from advanced health informatics applications involving DL. Despite astonishing advances in biomedical data analysis through ML and DL applications for novel biomarkers and therapeutic target identification, much work remains to be done to develop more effective and personalized treatments, through the exploitation of integrated data [51]. Big data analytics in the biomedical field, especially in ND research, is providing promising opportunities as shown by the growing initiatives of data sharing and standardized integration of multiple sources of information described in Section 5 and Section 6. DL can be used in a precision medicine framework and will be crucial to identify novel therapeutic targets and early biomarkers for diagnosis and improve clinical management for patients with complex and heterogeneous ND.

## Figures and Tables

**Table 1:** Summary of influential DL architectures and approaches for multi-layer big data analysis.

Architecture	Description	Graph
Deep Neural Network (DNN)	The basic network is made of multiple hidden layers. It is capable of modeling complex non-linear relationships by learning input data representation to be matched with a specific output [19].	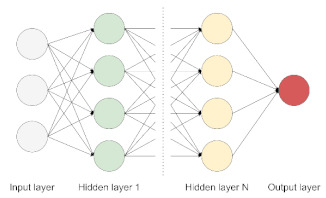
Autoencoder (AE)	It allows detecting patterns in the data in an unsupervised fashion. The model is made of an encoder and a decoder, transforming input data to generate its own representation, aiming to minimize the difference between the input and its output representation [20].	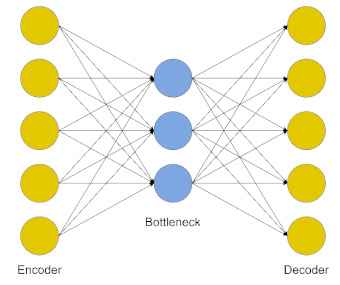
Restricted Boltzmann Machine (RBM)	This model is made of two layers, where nodes are bidirectionally connected but there are no connections within one layer. It is trained to learn a probability distribution for the input data and can be used as a building block for deep probabilistic models, where multiple RBMs can be stacked to build a deeper network [21].	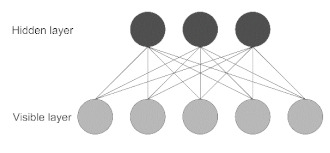
Convolutional Neural Network (CNN)	Most used for image processing in computer vision applications. The network uses convolution and pooling operations to extract relevant features from data, useful for image classification. This architecture is inspired by the organization of the visual cortex [22].	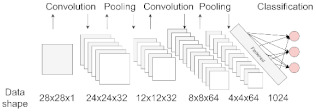
Recurrent Neural Network (RNN)	Best suited to process sequential data and used to predict the future from the past. The network can give an output for every timestep and takes the previous inputs into account to determine the output. Long-Short Term Memory (LSTM) and Gated Recurrent Units (GRUs) are RNN architectures [19].	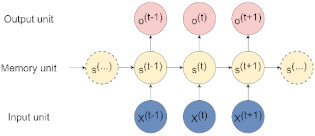

**Table 2:** Most representative databases, including data for ND research.

Database Name	ND	URL	Data Type	Description
PDGene	PD	http://www.pdgene.org, accessed on 19 February 2021	Omics	PDGene is a database providing results for potential risk loci in PD [61].
PPMI	PD	https://www.ppmi-info.org, accessed on 19 February 2021	Mixed	The Parkinson’s Progression Markers Initiative holds a comprehensive set of clinical, imaging, and biosample data to define biomarkers of PD progression [25].
NIAGADS	AD	https://www.niagads.org, accessed on 19 February 2021	Omics	The National Institute on Aging Genetics of Alzheimer’s Disease Data Storage Site is a repository that collects and shares genotypic data for the study of AD and related dementias [58].
ADNI	AD	http://adni.loni.usc.edu, accessed on 19 February 2021	Mixed	The Alzheimer’s Disease Neuroimaging Initiative is a multisite study for the prevention and treatment of AD. Its database stores a collection of validated study data to define the progression of AD, including mild cognitive impairment subjects and elderly controls [60].
NACC	AD	https://www.alz.washington.edu, accessed on 19 February 2021	Clinical	The National Alzheimer’s Coordinating Center holds a large relational database of standardized clinical and neuropathological research data for both exploratory and explanatory AD research [65].
LAADC	AD	https://www.ohsu.edu/brain-institute/clinical-data-resources, accessed on 19 February 2021	Clinical	Longitudinal relational database from the Layton Aging and Alzheimer’s Disease Center holding clinical data for over 4000 research subjects.
GEO	Mixed	http://www.ncbi.nlm.nih.gov/geo, accessed on 19 February 2021	Omics	Gene Expression Omnibus is a public functional genomics data repository of array-and sequence-based data [62].
UK Biobank	Mixed	https://www.ukbiobank.ac.uk, accessed on 19 February 2021	Omics	UK Biobank contains data from a large prospective study with over 500,000 participants and it aims to improve the prevention, diagnosis, and treatment of various illnesses, including dementia [63].
OmicsDI	Mixed	https://www.omicsdi.org/, accessed on 19 February 2021	Omics	Omics Discovery Index facilitates access to omics datasets from multiple studies through an integrated and open-source platform [57].
JPND	Mixed	https://www.neurodegenerationresearch.eu, accessed on 19 February 2021	Mixed	The Joint Programme Neurodegenerative Disease Research Database contains data from research related to neurodegenerative diseases from 27 member countries.
GAAIN	Mixed	http://www.gaaindata.org, accessed on 19 February 2021	Mixed	The Global Alzheimer’s Association Interactive Network is an online integrated research platform affiliated with partners all over the world, providing resources and data enabling comparative data analysis and cohort discovery [59].
Bio FINDER	Mixed	https://biofinder.se/, accessed on 19 February 2021	Mixed	The Swedish Biomarkers for Identifying Neurodegenerative Disorders Early and Reliably study aims to develop early diagnostic tests to identify novel treatment targets and understand the links between different ND and clinical symptoms.

## References

[1] Katsnelson A., De Strooper B., Zoghbi H.Y. (2016). Neurodegeneration: From cellular concepts to clinical applications. Sci. Transl. Med..

[2] Bovolenta T.M., de Azevedo Silva S.M.C., Arb Saba R., Borges V., Ferraz H.B., Felicio A.C. (2017). Systematic Review and Critical Analysis of Cost Studies Associated with Parkinson’s Disease. Park. Dis..

[3] Erkkinen M.G., Kim M.-O., Geschwind M.D. (2018). Clinical Neurology and Epidemiology of the Major Neurodegenerative Diseases. Cold Spring Harb. Perspect. Biol..

[4] Strafella C., Caputo V., Galota M.R., Zampatti S., Marella G., Mauriello S., Cascella R., Giardina E. (2018). Application of Precision Medicine in Neurodegenerative Diseases. Front. Neurol..

[5] Alexander N., Alexander D.C., Barkhof F., Denaxas S. (2020). Using Unsupervised Learning to Identify Clinical Subtypes of Alzheimer’s Disease in Electronic Health Records. Stud. Health Technol. Inform..

[6] Dujardin S., Commins C., Lathuiliere A., Beerepoot P., Fernandes A.R., Kamath T.V., De Los Santos M.B., Klickstein N., Corjuc D.L., Corjuc B.T. (2020). Tau molecular diversity contributes to clinical heterogeneity in Alzheimer’s disease. Nat. Med..

[7] Maudsley S., Devanarayan V., Martin B., Geerts H. (2018). Brain Health Modeling Initiative (BHMI) Intelligent and effective informatic deconvolution of “Big Data” and its future impact on the quantitative nature of neurodegenerative disease therapy. Alzheimers Dement. J. Alzheimers Assoc..

[8] Young A.L., Marinescu R.V., Oxtoby N.P., Bocchetta M., Yong K., Firth N.C., Cash D.M., Thomas D.L., Dick K.M., Cardoso J. (2018). Uncovering the heterogeneity and temporal complexity of neurodegenerative diseases with Subtype and Stage Inference. Nat. Commun..

[9] Manzoni C., Lewis P.A., Ferrari R. (2020). Network Analysis for Complex Neurodegenerative Diseases. Curr. Genet. Med. Rep..

[10] Dorsey E.R., Elbaz A., Nichols E., Abd-Allah F., Abdelalim A., Adsuar J.C., Ansha M.G., Brayne C., Choi J.-Y.J., Collado-Mateo D. (2018). Global, regional, and national burden of Parkinson’s disease, 1990–2016: A systematic analysis for the Global Burden of Disease Study 2016. Lancet Neurol..

[11] Nalls M.A., Blauwendraat C., Vallerga C.L., Heilbron K., Bandres-Ciga S., Chang D., Tan M., Kia D.A., Noyce A.J., Xue A. (2019). Identification of novel risk loci, causal insights, and heritable risk for Parkinson’s disease: A meta-analysis of genome-wide association studies. Lancet Neurol..

[12] De Jager P.L., Yang H.-S., Bennett D.A. (2018). Deconstructing and targeting the genomic architecture of human neurodegeneration. Nat. Neurosci..

[13] Docampo E., Giardina E., Riveira-Muñoz E., De Cid R., Escaramís G., Perricone C., Fernández-Sueiro J.L., Maymó J., González-Gay M.A., Blanco F.J. (2011). Deletion of LCE3C and LCE3B is a susceptibility factor for psoriatic arthritis: A study in Spanish and Italian populations and meta-analysis. Arthritis Rheum..

[14] Stocchi L., Cascella R., Zampatti S., Pirazzoli A., Novelli G., Giardina E. (2012). The pharmacogenomic HLA biomarker associated to adverse abacavir reactions: Comparative analysis of different genotyping methods. Curr. Genom..

[15] Arle J.E., Carlson K.W. (2021). Medical diagnosis and treatment is NP-complete. J. Exp. Theor. Artif. Intell..

[16] Camacho D.M., Collins K.M., Powers R.K., Costello J.C., Collins J.J. (2018). Next-Generation Machine Learning for Biological Networks. Cell.

[17] Perakakis N., Yazdani A., Karniadakis G.E., Mantzoros C. (2018). Omics, big data and machine learning as tools to propel understanding of biological mechanisms and to discover novel diagnostics and therapeutics. Metabolism.

[18] Valliani A.A.-A., Ranti D., Oermann E.K. (2019). Deep Learning and Neurology: A Systematic Review. Neurol. Ther..

[19] Goodfellow I., Bengio Y., Courville A. (2016). Deep Learning.

[20] Hinton G.E., Salakhutdinov R.R. (2006). Reducing the dimensionality of data with neural networks. Science.

[21] Hinton G.E. (2012). A practical guide to training restricted Boltzmann machines. Neural Networks: Tricks of the Trade.

[22] LeCun Y., Bottou L., Bengio Y., Haffner P. (1998). Gradient-based learning applied to document recognition. Proc. IEEE.

[23] Grapov D., Fahrmann J., Wanichthanarak K., Khoomrung S. (2018). Rise of Deep Learning for Genomic, Proteomic, and Metabolomic Data Integration in Precision Medicine. Omics J. Integr. Biol..

[24] Ramzan F., Khan M.U.G., Rehmat A., Iqbal S., Saba T., Rehman A., Mehmood Z. (2019). A Deep Learning Approach for Automated Diagnosis and Multi-Class Classification of Alzheimer’s Disease Stages Using Resting-State fMRI and Residual Neural Networks. J. Med. Syst..

[25] Marek K., Jennings D., Lasch S., Siderowf A., Tanner C., Simuni T., Coffey C., Kieburtz K., Flagg E., Chowdhury S. (2011). The Parkinson Progression Marker Initiative (PPMI). Prog. Neurobiol..

[26] Chakraborty S., Aich S., Kim H.-C. (2020). Detection of Parkinson’s Disease from 3T T1 Weighted MRI Scans Using 3D Convolutional Neural Network. Diagnostics.

[27] Hesamian M.H., Jia W., He X., Kennedy P. (2019). Deep Learning Techniques for Medical Image Segmentation: Achievements and Challenges. J. Digit. Imaging.

[28] Basher A., Kim B.C., Lee K.H., Jung H.Y. (2020). Automatic Localization and Discrete Volume Measurements of Hippocampi From MRI Data Using a Convolutional Neural Network. IEEE Access.

[29] Kushibar K., Valverde S., González-Villà S., Bernal J., Cabezas M., Oliver A., Lladó X. (2018). Automated sub-cortical brain structure segmentation combining spatial and deep convolutional features. Med. Image Anal..

[30] Brusini I., Lindberg O., Muehlboeck J., Smedby Ö., Westman E., Wang C. (2020). Shape information improves the cross-cohort performance of deep learning-based segmentation of the hippocampus. Front. Neurosci..

[31] Li F., Liu M., Initiative A.D.N. (2018). Alzheimer’s disease diagnosis based on multiple cluster dense convolutional networks. Comput. Med. Imaging Graph..

[32] Jain R., Jain N., Aggarwal A., Hemanth D.J. (2019). Convolutional neural network based Alzheimer’s disease classification from magnetic resonance brain images. Cogn. Syst. Res..

[33] Pereira C.R., Pereira D.R., Rosa G.H., Albuquerque V.H.C., Weber S.A.T., Hook C., Papa J.P. (2018). Handwritten dynamics assessment through convolutional neural networks: An application to Parkinson’s disease identification. Artif. Intell. Med..

[34] Drotár P., Mekyska J., Rektorová I., Masarová L., Smékal Z., Faundez-Zanuy M. (2015). Decision Support Framework for Parkinson’s Disease Based on Novel Handwriting Markers. IEEE Trans. Neural Syst. Rehabil. Eng..

[35] Pereira C.R., Weber S.A.T., Hook C., Rosa G.H., Papa J.P. Deep Learning-Aided Parkinson’s Disease Diagnosis from Handwritten Dynamics. Proceedings of the 2016 29th SIBGRAPI Conference on Graphics, Patterns and Images (SIBGRAPI).

[36] Berus L., Klancnik S., Brezocnik M., Ficko M. (2018). Classifying Parkinson’s Disease Based on Acoustic Measures Using Artificial Neural Networks. Sensors.

[37] Al-Hameed S., Benaissa M., Christensen H., Mirheidari B., Blackburn D., Reuber M. (2019). A new diagnostic approach for the identification of patients with neurodegenerative cognitive complaints. PLoS ONE.

[38] Camps J., Samà A., Martín M., Rodríguez-Martín D., Pérez-López C., Moreno Arostegui J.M., Cabestany J., Català A., Alcaine S., Mestre B. (2018). Deep learning for freezing of gait detection in Parkinson’s disease patients in their homes using a waist-worn inertial measurement unit. Knowl. Based Syst..

[39] Perez-Riverol Y., Zorin A., Dass G., Vu M.-T., Xu P., Glont M., Vizcaíno J.A., Jarnuczak A.F., Petryszak R., Ping P. (2019). Quantifying the impact of public omics data. Nat. Commun..

[40] Sonawane A.R., Weiss S.T., Glass K., Sharma A. (2019). Network Medicine in the Age of Biomedical Big Data. Front. Genet..

[41] Li Y., Wu F.-X., Ngom A. (2018). A review on machine learning principles for multi-view biological data integration. Brief. Bioinform..

[42] Espay A.J., Schwarzschild M.A., Tanner C.M., Fernandez H.H., Simon D.K., Leverenz J.B., Merola A., Chen-Plotkin A., Brundin P., Kauffman M.A. (2017). Biomarker-driven phenotyping in Parkinson’s disease: A translational missing link in disease-modifying clinical trials. Mov. Disord. Off. J. Mov. Disord. Soc..

[43] Ranchal R., Bastide P., Wang X., Gkoulalas-Divanis A., Mehra M., Bakthavachalam S., Lei H., Mohindra A. (2020). Disrupting Healthcare Silos: Addressing Data Volume, Velocity and Variety with a Cloud-Native Healthcare Data Ingestion Service. IEEE J. Biomed. Health Inform..

[44] Johnson A.E.W., Pollard T.J., Shen L., Lehman L.H., Feng M., Ghassemi M., Moody B., Szolovits P., Anthony Celi L., Mark R.G. (2016). MIMIC-III, a freely accessible critical care database. Sci. Data.

[45] Hernandez-Boussard T., Monda K.L., Crespo B.C., Riskin D. (2019). Real world evidence in cardiovascular medicine: Ensuring data validity in electronic health record-based studies. J. Am. Med. Inform. Assoc..

[46] Bender D., Sartipi K. HL7 FHIR: An Agile and RESTful approach to healthcare information exchange. Proceedings of the 26th IEEE international symposium on computer-based medical systems, Porto, Portugal, 20–22 June 2013.

[47] Kaur J., Mann K.S. (2017). AI based healthcare platform for real time, predictive and prescriptive analytics using reactive programming. Journal of Physics: Conference Series, Proceedings of the 10th International Conference on Computer and Electrical Engineering, Edmonton, AB, Canada, 11–13 October 2017.

[48] Lee C.S., Lee A.Y. (2020). Clinical applications of continual learning machine learning. Lancet Digit. Health.

[49] Goodstein D. (2009). Defining the scientific method. Nat. Methods.

[50] Ching T., Himmelstein D.S., Beaulieu-Jones B.K., Kalinin A.A., Do B.T., Way G.P., Ferrero E., Agapow P.-M., Zietz M., Hoffman M.M. (2018). Opportunities and obstacles for deep learning in biology and medicine. J. R. Soc. Interface.

[51] Zhavoronkov A., Mamoshina P., Vanhaelen Q., Scheibye-Knudsen M., Moskalev A., Aliper A. (2019). Artificial intelligence for aging and longevity research: Recent advances and perspectives. Ageing Res. Rev..

[52] Zhang X., Chou J., Liang J., Xiao C., Zhao Y., Sarva H., Henchcliffe C., Wang F. (2019). Data-Driven Subtyping of Parkinson’s Disease Using Longitudinal Clinical Records: A Cohort Study. Sci. Rep..

[53] Lee G., Nho K., Kang B., Sohn K.-A., Kim D. (2019). Predicting Alzheimer’s disease progression using multi-modal deep learning approach. Sci. Rep..

[54] Park C., Ha J., Park S. (2020). Prediction of Alzheimer’s disease based on deep neural network by integrating gene expression and DNA methylation dataset. Expert Syst. Appl..

[55] Xu J., Wu P., Chen Y., Meng Q., Dawood H., Dawood H. (2019). A hierarchical integration deep flexible neural forest framework for cancer subtype classification by integrating multi-omics data. BMC Bioinform..

[56] Gligorijević V., Malod-Dognin N., Pržulj N. (2016). Integrative methods for analyzing big data in precision medicine. Proteomics.

[57] Perez-Riverol Y., Bai M., da Veiga Leprevost F., Squizzato S., Park Y.M., Haug K., Carroll A.J., Spalding D., Paschall J., Wang M. (2017). Discovering and linking public omics data sets using the Omics Discovery Index. Nat. Biotechnol..

[58] Kuzma A., Valladares O., Cweibel R., Greenfest-Allen E., Childress D.M., Malamon J., Gangadharan P., Zhao Y., Qu L., Leung Y.Y. (2016). NIAGADS: The NIA Genetics of Alzheimer’s Disease Data Storage Site. Alzheimers Dement..

[59] Toga A.W., Neu S.C., Bhatt P., Crawford K.L., Ashish N. (2016). The Global Alzheimer’s Association Interactive Network. Alzheimers Dement. J. Alzheimers Assoc..

[60] Mueller S.G., Weiner M.W., Thal L.J., Petersen R.C., Jack C., Jagust W., Trojanowski J.Q., Toga A.W., Beckett L. (2005). The Alzheimer’s disease neuroimaging initiative. Neuroimaging Clin..

[61] Lill C.M., Roehr J.T., McQueen M.B., Kavvoura F.K., Bagade S., Schjeide B.-M.M., Schjeide L.M., Meissner E., Zauft U., Allen N.C. (2012). Comprehensive research synopsis and systematic meta-analyses in Parkinson’s disease genetics: The PDGene database. PLoS Genet..

[62] Edgar R., Domrachev M., Lash A.E. (2002). Gene Expression Omnibus: NCBI gene expression and hybridization array data repository. Nucleic Acids Res..

[63] Sudlow C., Gallacher J., Allen N., Beral V., Burton P., Danesh J., Downey P., Elliott P., Green J., Landray M. (2015). UK Biobank: An Open Access Resource for Identifying the Causes of a Wide Range of Complex Diseases of Middle and Old Age. PLOS Med..

[64] Giardina E., Caltagirone C. (2018). The IRCCS Network of Neuroscience and Neurorehabilitation: The Italian Platform for Care and Research about Neurodegenerative Disorders. Eur. J. Neurol..

[65] Beekly D.L., Ramos E.M., Lee W.W., Deitrich W.D., Jacka M.E., Wu J., Hubbard J.L., Koepsell T.D., Morris J.C., Kukull W.A. (2007). The National Alzheimer’s Coordinating Center (NACC) Database: The Uniform Data Set. Alzheimer Dis. Assoc. Disord..

[66] Leff D.R., Yang G.-Z. (2015). Big Data for Precision Medicine. Engineering.

[67] Khoury M.J., Iademarco M.F., Riley W.T. (2016). Precision Public Health for the Era of Precision Medicine. Am. J. Prev. Med..

[68] Zhou L., Verstreken P. (2018). Reprogramming neurodegeneration in the big data era. Curr. Opin. Neurobiol..

[69] Barabási A.-L., Gulbahce N., Loscalzo J. (2011). Network medicine: A network-based approach to human disease. Nat. Rev. Genet..

[70] Huang S., Chaudhary K., Garmire L.X. (2017). More is better: Recent progress in multi-omics data integration methods. Front. Genet..

[71] Sethi A., Sankaran A., Panwar N., Khare S., Mani S. (2017). DLPaper2Code: Auto-generation of Code from Deep Learning Research Papers. arXiv.

[72] Sammut C., Webb G.I. (2011). Encyclopedia of Machine Learning.

[73] Lever J., Krzywinski M., Altman N. (2016). Regularization.

[74] Molina L.C., Belanche L., Nebot À. Feature selection algorithms: A survey and experimental evaluation. Proceedings of the 2002 IEEE International Conference on Data Mining, 2002. Proceedings.

[75] Gibney E. (2016). Google AI algorithm masters ancient game of Go. Nat. News.

[76] Lundberg S.M., Lee S.-I. (2017). A Unified Approach to Interpreting Model Predictions. Adv. Neural Inf. Process. Syst..

[77] Selvaraju R.R., Cogswell M., Das A., Vedantam R., Parikh D., Batra D. Grad-cam: Visual explanations from deep networks via gradient-based localization. Proceedings of the IEEE International Conference on Computer Vision.

[78] Singh A., Sengupta S., Lakshminarayanan V. (2020). Explainable Deep Learning Models in Medical Image Analysis. J. Imaging.

[79] Sharma A., Shukla D., Goel T., Mandal P.K. (2019). BHARAT: An Integrated Big Data Analytic Model for Early Diagnostic Biomarker of Alzheimer’s Disease. Front. Neurol..

